# Effect of Opposite Tooth Condition on Marginal Bone Loss around Submerged Dental Implants: A Retrospective Study with a 3-Year Follow-Up

**DOI:** 10.3390/ijerph182010715

**Published:** 2021-10-13

**Authors:** Odontuya Dorj, Hsi-Kuei Lin, Eisner Salamanca, Yu-Hwa Pan, Yi-Fan Wu, Yung-Szu Hsu, Jerry C-Y Lin, Chin-Kai Lin, Wei-Jen Chang

**Affiliations:** 1 School of Dentistry, College of Oral Medicine, Taipei Medical University, Taipei 11031, Taiwan; dorj.odontuya@gmail.com (O.D.); linhsikuei38@gmail.com (H.-K.L.); eisnergab@hotmail.com (E.S.); shalom.dc@msa.hinet.net (Y.-H.P.); yfwu@tmu.edu.tw (Y.-F.W.); nm8346@yahoo.com.tw (Y.-S.H.); drjerrylin@gmail.com (J.C.-Y.L.); 2 Department of Dental technology and Dental Hygiene, School of Dentistry, Mongolian National University of Medical Sciences, Ulaanbaatar 14210, Mongolia; 3 School of Oral Hygiene, College of Oral Medicine, Taipei Medical University, Taipei 10692, Taiwan; 4 Dental Department, Taipei Medical University, Shuang-Ho Hospital, New Taipei City 23561, Taiwan; 5 Department of Dentistry, Chang Gung Memorial Hospital, Taipei 83301, Taiwan; 6 Graduate Institute of Dental & Craniofacial Science, Chang Gung University, Taoyuan 33305, Taiwan; 7 School of Dentistry, College of Medicine, China Medical University, Taichung 40402, Taiwan; 8 Department of Oral Medicine, Infection and Immunity, Harvard School of Dental Medicine, Boston, MA 02115, USA; 9 Department of Dentistry, En Chu Kong Hospital, New Taipei City 237, Taiwan

**Keywords:** submerged dental implants, marginal bone level, opposing condition, natural teeth, fixed restoration

## Abstract

Background: The objective of this study was to evaluate the effects of opposite tooth conditions on change in marginal bone level (MBL) around submerged dental implants. Materials and methods: The study included healthy patients with one or two implants. Structures opposite implants were either natural teeth (NT) or fixed restorations (FRs). MBLs were measured on digital periapical radiographs at the mesial and distal aspects of each implant. Results: Sixty implants were inserted by the 3-year follow-up. Mean MBLs for NT were 0.21 ± 0.33 mm before prosthetic loading and 0.30 ± 0.41 mm 3 years later (*p* = 0.001). Mean MBLs with FRs were 0.36 ± 0.45 mm before loading and 0.53 ± 0.50 mm 3 years later (*p* < 0.001). Changes in mean MBL from the 6-month follow-up to the 1- and 3-year follow-ups were statistically significant (*p* < 0.01) for implants opposite NT. However, changes in mean MBL from the 6-month follow-up to the 1-year (*p* = 0.161) and 3-year follow-ups (*p* = 1.000) were not significant for implants opposite FRs. Between baseline and the 3-year follow-up, MBL change was relatively small and did not differ regarding NT and FRs. Conclusion: Bone loss was greater if submerged dental implants were opposed by FRs. MBLs around submerged implants continued to change after 3 years if NT opposed implants.

## 1. Introduction

Marginal bone alteration and osseointegrated dental implants that support a variety of prostheses have enhanced oral rehabilitation. Clinical and radiographic measurements of dental implants and their suprastructures must be assessed to identify treatment success and failure determinants.

Marginal bone around dental implants undergoes remodeling and resorption after implant placement [1]. According to modified criteria of success for dental implant systems, average crestal bone loss should be less than 1.5 mm during the first year, and subsequent annual bone loss should be less than 0.2 mm [2]. Examination of radiographic crestal bone level is crucial for determining the long-term success of periimplant health [3].

Noticeable marginal bone changes during implant placement and abutment connection and at the beginning of functional loading have occurred, and minor bone loss has been observed [4]. In an experimental study in dogs, functional loading at implants increased osseointegration and did not induce marginal bone loss [5]. In another study of crestal bone changes around submerged implants in the canine mandible, bone remodeling occurred after abutment connection. The rough–smooth border of the implant surface significantly affected marginal bone alteration [6]. Radiographic evaluation of crestal bone levels revealed that crestal bone loss around nonsubmerged implants up to 5 years after loading was minor. The factors that affected marginal bone remodeling in the early and later implant placement were significantly different [7].

Dental implant modifications were initiated to enhance the osseointegration procedure [8,9]. Because periimplant bone loss is induced by mechanical factors such as occlusal overload or biological reasons, dental implant design remains a risk factor for failure [10,11,12,13,14]. The quantity of strength and the direction of the natural occlusal forces are hard to quantify [15]. In addition, the crestal bone demonstrates varying tolerance, depending on its location and biological and anatomical parameters. In animal studies, functional loading of the dental implants improved the remodeling process around implant-bone tissue, and osseointegration could be regained once the excessive load is removed from the occlusion [10]. Mechanical stress transferred to the surrounding bone of the implant is clearly the key to successful osseointegration and its long existence [16]. Osseointegration incorporates many biological activities that are affected by various factors. The design of dental implants can optimize the occlusal load transfer and have an effect on final osseointegration. Characteristics of implant material, such as low elastic modulus and high porosity, have proved advantageous for osseointegration in several studies, but their effects in total replacement remain unclear [17].

Structural disparities between implants and natural teeth (NT) affect the transmission of force to the bone. According to two reports, implant design affected strain distribution around opposing NT, and the magnitudes of strain around the natural tooth opposite an antagonist implant were significantly less than those around the occluding implants [18,19]. The type of opposing structures has been related to alterations in crestal bone levels around dental implants. In a study of various local and systemic effects on periimplant bone levels around implants with a single tooth–locking tapered design, a significant relation was found between the type of opposing structure and the alteration in crestal bone levels. The bone gain was reported when the opposing structure was a natural tooth, whereas bone loss increase was noted around implants opposing implant-supported prostheses [20]. In contrast, increased bone loss after 1 year of loading was observed around 94 implants when opposed by either NT or fixed prostheses [21]. In a review study with a follow-up period of at least 3 years, no implant system showed superiority in marginal bone preservation [1]. A meta-analysis revealed that marginal bone level (MBL) differed significantly between different implant systems after 5-year functional loading [22].

Therefore, the possible effects of specific implant characteristics on periimplant bone alteration under different opposing conditions need to be clarified. We hypothesized that marginal bone loss around a natural tooth opposing a dental implant would be lower than that around an opposing fixed prosthesis. The purpose of this study was to evaluate the effects of opposing conditions on marginal bone loss around submerged dental implants over a 3-year follow-up period.

## 2. Materials and Methods

**Figure 1:**
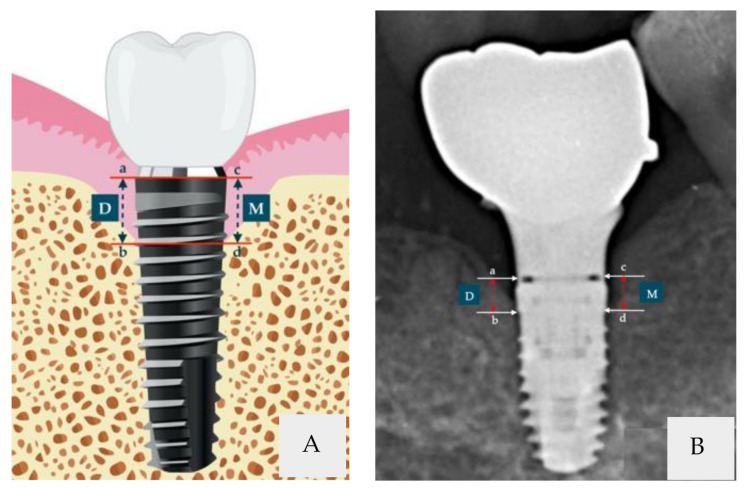
Schematic illustration of submerged dental implants. (**A**) Vector illustration, (**B**) X-ray image. The marginal bone level was measured from the rough–smooth border of the implant surface to the alveolar crest on the mesial and distal aspects of the tooth. D, length of the distal surface (a–b); M, length of the mesial surface (c–d).

In this retrospective study, the data of the patients were obtained from the Shuang-Ho Hospital, New Taipei, Taiwan, between October 2008 and March 2020. The Joint Institutional Review Board approved the study protocol of Taipei Medical University (registration number N202103105). The same oral surgeon and prosthodontist performed all surgical and prosthetic procedures. A total of 60 submerged dental implants were inserted in 46 patients (Figure 1). All 60 implants were opposed by either NT (the NT group) or fixed restorations (FRs; FR group), which included implant teeth (IT) and fixed partial prostheses (FPPs). Each patient, who was monitored for up to 3 years, received one or two implants. Radiographic parameters were evaluated at baseline (before loading), immediately after prosthetic loading, and 3 months, 6 months, 1 year, and 3 years after loading. The following inclusion criteria were used: >18 years of age who received one dental implant, non-smokers, absence of systemic diseases, having a good oral hygiene, no active inflammation, adequate bone tissue to ensure primary implant stability, and keratinized tissue >2 mm at the time of implant placement. The following exclusion criteria were used: Patients with presence of immune and infectious diseases, radiotherapy or other types of cancer treatments 12 months prior to study, presence of uncontrolled diabetes, betel nut chewing habit, pregnant or breastfeeding patients, bone augmentation procedures, and unwillingness to return for follow-up radiography. 

The implants employed in this study, Xive submerged dental implants (Dentsply Sirona, Mannheim, Germany), were positioned in the posterior area (e.g., as second premolars and first and second molars). The implants were 3.8 and 4.5 mm in diameter and 9.5 and 11 mm long. MBLs were measured with EZ Dental professional image software (charged-couple device digital X-ray system, Hyper-X CM). The lengths from the rough–smooth border of implant surfaces to the highest MBL were measured, and an average mesiodistal bone level was calculated for each implant. [23] The standard long-cone paralleling technique, used in each periapical radiograph and X-ray cone indicator, was performed to evaluate the bite on the film. The EZ Dental software calibration tool repaired the deviation in periapical films. A positive value was adopted if the alveolar crest was above the rough–smooth junction. The value was zero if the alveolar crest level paralleled the rough–smooth junction. The value was negative if the bone crest was below the rough–smooth junction. The percentage of bone-to-implant contact (BIC) of the submerged dental implants were analyzed. BIC indicates the percentage of implant surface in direct connection with the bone over the total length of the implant. 

### Statistical Methods

The chi-square and independent *t*-tests were used to compare the baseline data—age, gender, implant diameters, lengths, and locations—of the NT and FR groups. A paired *t*-test compared the MBLs and other measurements of the NT and FR groups. An independent *t*-test analyzed the two groups. A *p*-value of <0.05 indicated statistical significance. We used SPSS software (IBM, Armonk, NY, USA) to perform the analyses.

## 3. Results

### 3.1. Patient Characteristics at Baseline

A total of 60 submerged dental implants were inserted in 46 patients (25 men and 21 women). The patients’ average age was 50.3 years. The diameter of the inserted implants ranged from 3.8 to 4.5 mm, and the lengths were 9.5 and 11 mm. Of the 60 implants, 29 (48%) were placed in the upper jaw, and 31 (52%) were placed in the lower jaw (Table 1). The data about structures (NT and FR) opposing the implants were compared at baseline (before loading), immediately after loading, and 3 months, 6 months, 1 year, and 3 years after loading. No implant failures occurred in either the NT or FR group, and no complications were reported during the follow-up periods.

### 3.2. Opposing Structures and Marginal Bone Level Analysis

**Figure 2:**
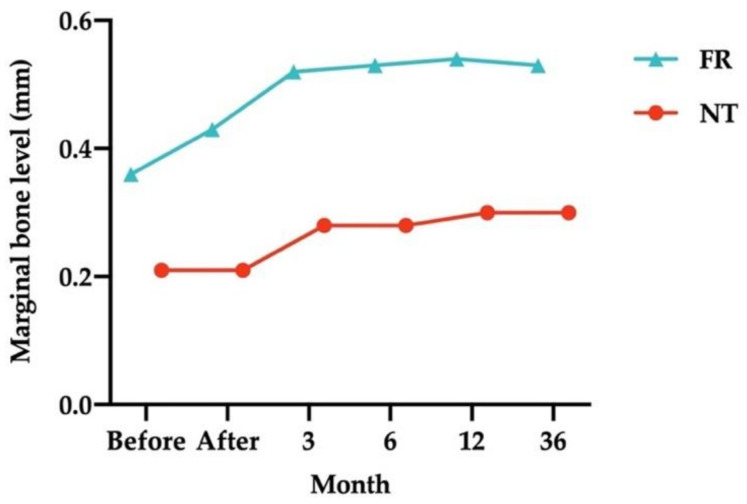
Mean marginal bone levels of the submerged dental implants opposed by natural teeth (NT) and fixed restorations (FRs) at all follow-up times. Measurements at the bottom of the graph refer to the time of implant loading.

Of the implants, 29 were opposed by NT, and implant-supported FRs opposed 31. Figure 2 illustrates the MBLs around the submerged dental implants at all follow-up times. The mean MBL of the submerged implants that NT opposed was lower than that of the implants opposed by FRs at all times.

MBLs of the submerged dental implants opposed by the NT and FRs are listed in Table 2a–b. In the NT group, MBLs remained the same between baseline (before loading) to immediately after loading (0.21 ± 0.33 mm). MBLs increased after 3 and 6 months (to 0.28 ± 0.39 mm) and after 1 and 3 years (to 0.30 ± 0.41 mm). These increases were significant at all follow-up times until the 3-year follow-up (*p* = 0.001). MBLs changed significantly from the 3-month follow-up to the 1-year (*p* = 0.003) and 3-year follow-ups (*p* = 0.005) and from the 6-month follow-up to the 1-year (*p* = 0.003) and 3-year follow-ups (*p* = 0.005). In the FR group, MBLs changed slightly from baseline (0.36 ± 0.45 mm) to immediately after loading (0.43 ± 0.48 mm; nonsignificant) and the 3-month (0.52 ± 0.50 mm; *p* = 0.001), 6-month (0.53 ± 0.50 mm; *p* = 0.001), 1-year (0.54 ± 0.52 mm; *p* < 0.001), and 3-year follow-ups (0.53 ± 0.50 mm; *p* < 0.001).

Table 3 lists changes in MBL over time. In the NT group, mean MBL did not change from baseline to immediately after loading (0.00 ± 0.00 mm); the mean change at the 3-month follow-up was significant (0.07 ± 0.11 mm; *p* = 0.001), but the MBLs remained the same at the 6-month follow-up. At the 1-year follow-up, MBLs changed significantly (to 0.09 ± 0.14 mm; *p* = 0.001) and remained the same at the 3-year follow-up. Similarly, the mean changes from the 3-month follow-up to the 1-year (*p* = 0.003) and 3-year follow-ups (*p* = 0.005) were significant, as were changes from the 6-month follow-up to the 1- and 3-year follow-ups. In the FR group, the MBL changed between baseline and immediately after prosthetic loading (to 0.07 ± 0.20 mm). Mean MBL changes were significant 3 months (0.16 ± 0.23 mm; *p* = 0.002), 6 months (0.17 ± 0.23 mm; *p* = 0.002), 1 year (0.18 ± 0.24 mm; *p* = 0.001), and 3 years (0.17 ± 0.22 mm; *p* = 0.001) after prosthetic loading.

**Figure 3:**
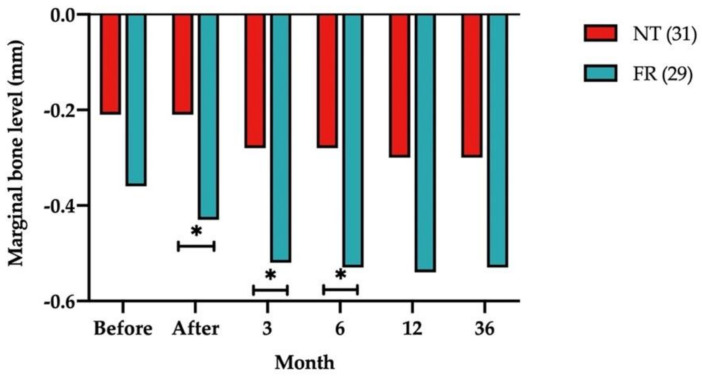
Comparison of mean marginal bone levels with natural teeth (NT) and fixed restorations (FRs) in the opposing arch group at all times. Numbers in parentheses indicate the numbers of NT and FRs. Measurements at the bottom of the graph refer to the time of implant loading. * *p* < 0.05.

Figure 3 illustrates the MBLs of submerged dental implants with NT and FRs in the opposing arches. MBL changed more with FRs than with NT at all time points. The mean MBL differences between the NT and FR groups were as follows: 0.22 ± 0.41 mm immediately after prosthetic loading (*p* = 0.047), 0.24 ± 0.45 mm at the 3-month follow-up (*p* = 0.041), and 0.25 ± 0.45 mm at the 6-month follow-up (*p* = 0.039).

**Figure 4:**
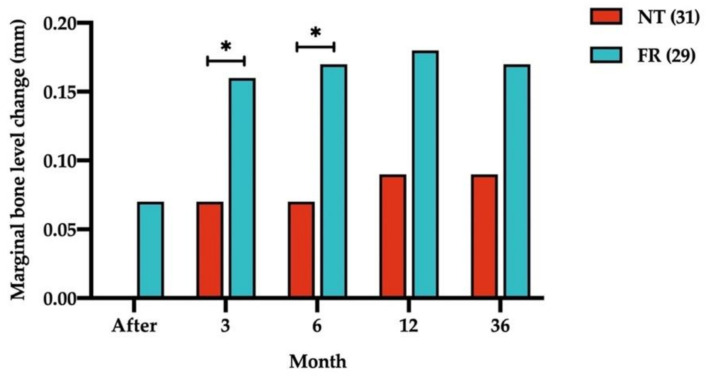
Comparison of change in mean marginal bone levels with natural teeth (NT) and fixed restorations (FRs) in the opposing arch group at all follow-up times. Numbers in parentheses indicate the numbers of NT and FRs. Measurements at the bottom of the graph refer to the time of implant loading. * *p* < 0.05.

Figure 4 illustrates MBL changes scores between NT and FR opposing arch groups. The mean MBL change differences between NT and FR groups were as follows: 0.09 ± 0.17 mm at the 3-month follow-up (*p* = 0.050) and 0.10 ± 0.17 mm at the 6-month follow-up (*p* = 0.046).

During the first year of follow-up, the average MBLs of submerged dental implants were 0.30 ± 0.40 mm with opposing NT and 0.54 ± 0.52 mm with opposing FRs; the differences were not statistically significant (*p* = 0.059). At the 3-year follow-up, MBLs were almost identical (0.30 ± 0.41 mm for NT and 0.53 ± 0.51 mm for FRs; *p* = 0.062). The corresponding MBL change during the first year was 0.09 ± 0.14 mm for NT and 0.18 ± 0.24 mm for FRs (*p* = 0.094). MBL changes at the 3-year follow-up were 0.09 ± 0.13 mm for NT and 0.17 ± 0.22 mm for FRs (*p* = 0.108).

MBLs and changes in MBLs for different types of FRs—FPPs and IT—are listed in Table 4. No significant difference was observed between FPPs and IT at any time.

### 3.3. Opposing Structures and Osseointegration Analysis

**Figure 5:**
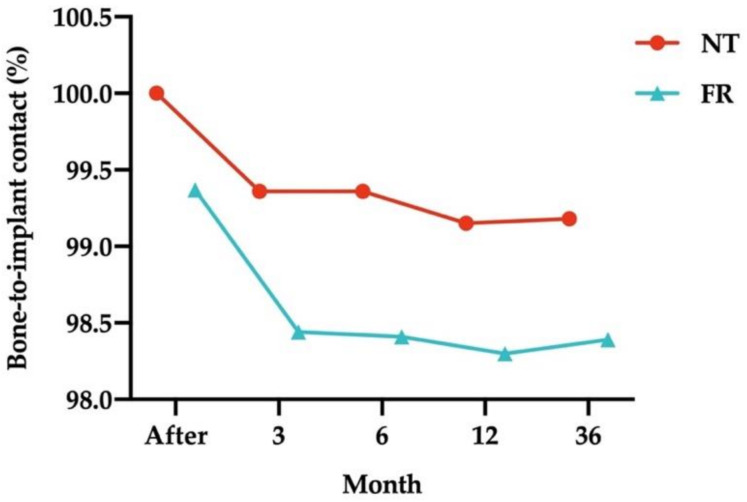
Mean percentages of bone-to-implant contact of the submerged dental implants opposed by natural teeth (NT) and fixed restorations (FRs) at all follow-up times. Measurements at the bottom of the graph refer to the time of implant loading.

Figure 5 illustrates the mean percentages of bone-to-implant contact (BIC) of the submerged dental implants at all times. Mean percentages of BIC for implants opposed by NT were higher than those for implants opposed by FRs over time.

Table 5 lists the percentages of BIC in the submerged dental implants opposed by NT and FRs. In the NT group, the mean percentages of BIC did not change from baseline to immediately after loading, but 3 months after loading, they did change significantly (99.36% ± 1.01%; *p* = 0.001) and then remained at the same level 6 months after loading. Mean percentages 1 year (99.15% ± 1.29%; *p* = 0.001) and 3 years (99.18% ± 1.28%; *p* = 0.001) after loading differed significantly. Similarly, mean percentages differed significantly between the 3-month follow-up and the 1-year (*p* = 0.003) and 3-year follow-ups (*p* = 0.005). The same differences were observed between the 6-month follow-up and the 1- and 3-year follow-ups. In the FR group, the mean percentages of BIC in the FR group were 99.37% ± 1.81% immediately after prosthetic loading (nonsignificant), 98.44% ± 2.15% at the 3-month follow-up (*p* = 0.002), 98.41% ± 2.19% at the 6-month follow-up (*p* = 0.002), 98.30% ± 2.22% at the 1-year follow-up (*p* = 0.001), and 98.39% ± 2.12% at the 3-year follow-up (*p* = 0.001).

**Figure 6:**
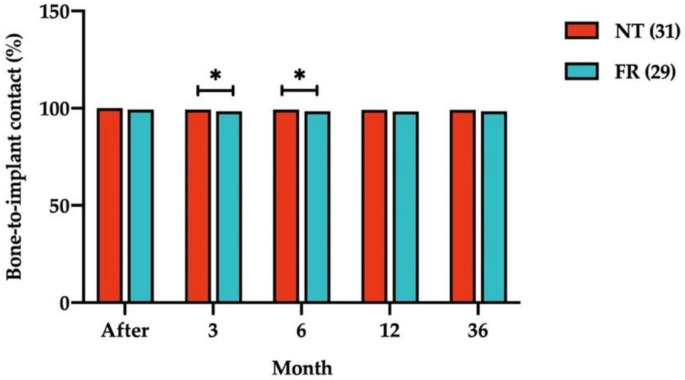
Comparison of percentage of bone-to-implant contact between natural teeth (NT) and fixed restorations (FRs) in the opposing arch at all follow-up times. Numbers in parentheses indicate the numbers of NT and FRs. Measurements at the bottom of the graph refer to the time of implant loading. * *p* < 0.05.

Figure 6 illustrates percentages of BIC for submerged dental implants between NT and FR opposing arch groups. These percentages were lower for implants opposed by FRs than for those opposed by NT at all times. However, the difference between percentages of BIC for the two groups remained minor. The mean differences in percentages of BIC between the NT and FR groups were −0.92% ± 1.58% (*p* = 0.043) at the 3-month follow-up and −0.95% ± 1.60% (*p* = 0.039) at the 6-month follow-up. Furthermore, percentages of BIC did not differ significantly between FPPs and IT at any time.

## 4. Discussion

Clinical information about the effects of opposing tooth conditions on MBL around dental implants is still limited. This study aimed to evaluate the effects of opposite tooth conditions on marginal bone loss around submerged dental implants. We hypothesized that submerged dental implants with opposing NT would show significantly less marginal bone loss over a three-year period than implants with opposing FRs. We found that MBLs for submerged dental implants that NT opposed were lower than those for implants opposed by FRs.

The outcomes of this study indicate that the different types of antagonist structure have a significant effect in MBL of the submerged dental implants. When the opposing structure was a FR, MBL was significantly lower, but 1 and 3 years after loading, the reduction was less than 1 mm. Conversely, opposing NT was associated with significantly less of a reduction in MBL. Of interest was that the MBLs of the submerged dental implants opposite NT were still changing 3 years after loading.

Urdaneta et al. evaluated periimplant bone loss around 310 single-tooth implants in an average follow-up period of 70.7 months. They found statistically significant differences between average changes in the bone levels of implants that were opposed by NT (0.20 mm) and those opposed by implant-supported restorations (0.62 mm) [24]. Boronat et al. reported periimplant bone loss around 106 implants 1 year after loading; the mean bone loss was 0.52 mm in the implants with opposing NT and 0.57 mm in the implants with opposing implant-supported prostheses [21]. In contrast, Carlsson et al. evaluated 273 smooth surface implants and found no differences in marginal bone loss between implants opposed by fixed implant-supported restorations and those opposed by complete dentures [25]. Our study revealed significantly less marginal bone loss around submerged dental implants that were opposed by NT than around those opposed by FRs, and the marginal bone loss values in our study were lower than those in the aforementioned studies.

A study of periimplant bone stress around implants demonstrated that the splinting of implants lessens periimplant bone stress by spreading the forces between the implants [26]. In a comparative study of the effect of splinting and interproximal point tightness on load transmission, the results suggested that splinting improved load transmission better than did nonsplinting [27]. Recent finite element analysis of the mechanical response of implant-supported dental restorations opposing different materials and abutments (implant and tooth) demonstrated that opposing implant-supported ceramic crowns had higher stress values than did other NT and acrylic all-on-4 plus titanium bars or NT plus ceramic crowns. However, that study did not reveal any difference in stresses weight in comparing NT with tooth-supported ceramic prostheses. The use of inflexible materials with higher elastic modulus may transmit more stress to the periimplant bone [28]. These findings may be elucidated by the notable differences between previously mentioned studies and our study. The most significant difference is that we evaluated the effects of opposing conditions on MBL around one or more implants. In contrast, in previous studies, the investigators evaluated the effects of opposing structures on either splinted or single-tooth implants with different surface coatings. Another difference is that we evaluated and compared the effects of two dissimilar opposing structures on submerged dental implants and demonstrated more marginal bone loss around implants that opposed FRs. Additionally, other differences, such as follow-up period, implant loading protocol, surface treatment, sample size, and implant design, may explain the variation in results.

In addition, we found that the MBLs of implants changed significantly over entire follow-up periods when NT opposed those implants, whereas those of implants that opposed FRs did not exhibit much change at the 3-month, 6-month, 1-year, and 3-year follow-ups. The change may be a biological reaction of the crestal bone to different mechanical forces generated by different opposing conditions during mastication. Our results are inconsistent with the findings of Urdaneta et al. [24] and Mericske-Stern et al. [29]. Urdaneta et al. reported increased bone levels around hydroxyapatite-coated mandibular implants opposing NT [24]. Mericske-Stern et al. evaluated the biting force on single implants and found that the implants opposing NT had more occlusal force than did those opposing FPPs, regardless of the design of the FR [29]. Thus, it is possible that the increased bone level around implants opposing NT was a response to the enhanced strain generated by the natural opposing condition during functional loading. Additionally, this increase may be explained by the use of different dental implant designs and the surface treatment used in these studies.

Some clinical studies of the effects of load on periimplant bone level around dental implants with rough surface treatments have shown contrasting results. Crestal bone level around a single-tooth implant is not related to the crown-to-implant ratio [30]. Different implant prosthetic modalities did not affect crestal bone loss around screw-retained nonsubmerged dental implants [31]. The opposing structures had a significant effect in different anatomical areas in the mouth, such as mandibular crestal bone, which indicates that peak strain conditions for bone exist in that area [32]. Prospective studies are needed to evaluate the long-term site-specific effects of opposing conditions on MBLs around implants with different designs. Excessive loading from the occluding is the main contributor to implant failure, and the load transmitted to the implant differs, depending on the occlusal relation to the opposing tooth. A finite element analysis revealed excessive stress at the interface between the implant and the abutment in cases of alveolar resorption with a substantial clearance between the implant and the opposing tooth, and this stress can be altered by implant inclination under conditions of horizontal contact between the implant and the opposing tooth [33].

According to modified criteria of success for dental implant systems, marginal bone loss averages less than 1.5 mm during the first year, and subsequent annual bone loss should be less than 0.2 mm [2]. In our study, marginal bone loss during the first year after loading was 0.30 mm for implants opposing NT and 0.54 mm for implants opposing FRs. The corresponding MBL changes by the 3-year follow-up were 0.09 and 0.17 mm, respectively. In other words, the implant system utilized in our study met the proposed criteria of success, although different structures opposed the implants. Our findings are consistent with data reported in some clinical studies [34,35].

The study’s limitations include the small sample size, the study of only one implant design, incompleteness of records (such as whether patients underwent endodontic treatment and periodontal clinical parameters), lack of information about opposing structure materials, and the use of two-dimensional radiographs. A larger sample size would have increased the power of this prospective study. Clinical studies with various dental implants and sizes in which different prosthetic restorations are compared with implant-supported restorations, in combination with finite element analysis, can provide a better understanding of bone remodeling in the future.

## 5. Conclusions

Within the limitations of this study, we derived the following conclusions:

1. Bone loss with submerged dental implants was more likely to occur if the implants were opposed by FRs (such as IT and FPPs).
2. MBL around submerged dental implants is still changing 3 years after loading if the implants are opposed by NT.

## Figures and Tables

**Table 1:** Patient and implant characteristics at baseline.

Parameters	Natural Teeth (*n* = 31)		Fixed Restorations (*n* = 29)		*p*-Value
Age (mean ± standard deviation)	49.2 ± 10.46		52.1 ± 6.49		0.252
Gender					0.685
Male	14	51.9%	11	57.9%	
Female	13	48.1%	8	42.1%	
Location					0.993
Upper jaw	15	48.4%	14	48.3%	
Lower jaw	16	51.6%	15	51.7%	
Diameter					0.769
3.8 mm	16	51.6%	11	37.9%	
4.5 mm	15	48.4%	18	62.1%	
Length					0.998
9.5 mm	11	35.5%	11	37.9%	
11 mm	20	64.5%	18	62.1%	

**Table 2:** (**a**) Analysis of marginal bone level with natural teeth in the opposing arch of the submerged dental implants. (**b**) Analysis of marginal bone level with fixed restorations in the opposing arch of the submerged dental implants.

(**a**)						
**Opposing Structure**	**Time (Relative to Loading)**	**Mean (mm)**	***p*-Value**			
			**Before Loading**	**3 M**	**6 M**	**12 M**
Natural tooth (*n* = 31)	Before	0.21 ± 0.33				
	Immediately after	0.21 ± 0.33	NA			
	3 months after	0.28 ± 0.39	0.001			
	6 months after	0.28 ± 0.39	0.001	NA		
	12 months after	0.30 ± 0.40	0.001	0.003	0.003	
	36 months after	0.30 ± 0.41	0.001	0.005	0.005	0.325
(**b**)						
**Opposing Structure**	**Time (Relative to Loading)**	**Mean (mm)**	***p*-Value**			
			**Before Loading**	**3 M**	**6 M**	**12 M**
Fixed restoration (*n* = 29)	Before	0.36 ± 0.45				
	Immediately after	0.43 ± 0.48	0.072			
	3 months after	0.52 ± 0.50	0.001			
	6 months after	0.53 ± 0.50	0.001	0.325		
	12 months after	0.54 ± 0.52	<0.001	0.029	0.161	
	36 months after	0.53 ± 0.51	<0.001	0.752	1.000	0.325

3 M, 3-month follow-up, 6 M, 6-month follow-up; 12 M, 12-month follow-up; NA, not applicable.

**Table 3:** Analysis of changes in marginal bone level with natural teeth and fixed restorations in the opposing arch of the submerged dental implants.

Opposing Structure	Time (Relative to Loading)	Mean (mm)	*p*-Value			
			Before Loading	3 M	6 M	12 M
Natural tooth (*n* = 31)	Immediately after	0.00 ± 0.00				
	3 months after	0.07 ± 0.11	0.001			
	6 months after	0.07 ± 0.11	0.001	NA		
	12 months after	0.09 ± 0.14	0.001	0.003	0.003	
	36 months after	0.09 ± 0.13	0.001	0.005	0.005	0.325
Fixed restoration (*n* = 29)	Immediately after	0.07 ± 0.20				
	3 months after	0.16 ± 0.23	0.002			
	6 months after	0.17 ± 0.23	0.002	0.325		
	12 months after	0.18 ± 0.24	0.001	0.029	0.161	
	36 months after	0.17 ± 0.22	0.001	0.752	1.000	0.325

3 M, 3-month follow-up; 6 M, 6-month follow-up; 12 M, 12-month follow-up; NA, not applicable.

**Table 4:** Analysis of MBL and change in MBL between FPPs and IT at all times.

Parameters	Time (Relative to Loading)	Mean (mm)		*p*-Value
		FPP (*n* = 15)	IT (*n* = 14)	
MBL	Before	0.26 ± 0.32	0.48 ± 0.55	0.209
	Immediately after	0.35 ± 0.36	0.52 ± 0.58	0.365
	3 months after	0.43 ± 0.38	0.63 ± 0.61	0.298
	6 months after	0.43 ± 0.38	0.64 ± 0.61	0.280
	12 months after	0.44 ± 0.38	0.64 ± 0.63	0.311
	36 months after	0.43 ± 0.37	0.63 ± 0.62	0.318
Change in MBL	Immediately after	0.09 ± 0.26	0.04 ± 0.11	0.496
	3 months after	0.17 ± 0.28	0.15 ± 0.17	0.849
	6 months after	0.17 ± 0.28	0.16 ± 0.19	0.916
	12 months after	0.18 ± 0.28	0.17 ± 0.18	0.862
	36 months after	0.18 ± 0.28	0.15 ± 0.14	0.782

FPP, fixed partial prosthesis; IT, implant tooth; MBL, marginal bone level.

**Table 5:** Analysis of percentage of bone-to-implant contact of natural teeth and fixed restorations in the opposing arch of the submerged dental implants.

Opposing Structure	Time (Relative to Loading)	Mean (%)	*p*-Value			
			Before Loading	3 M	6 M	12 M
Natural tooth (*n* = 31)	Immediately after	100.00 ± 0.00				
	3 months after	99.36 ± 1.01	0.001			
	6 months after	99.36 ± 1.01	0.001	NA		
	12 months after	99.15 ± 1.29	0.001	0.003	0.003	
	36 months after	99.18 ± 1.28	0.001	0.005	0.005	0.325
Fixed restoration (*n* = 29)	Immediately after	99.37 ± 1.81				
	3 months after	98.44 ± 2.15	0.002			
	6 months after	98.41 ± 2.19	0.002	0.325		
	12 months after	98.30 ± 2.22	0.001	0.029	0.134	
	36 months after	98.39 ± 2.12	0.001	0.752	0.880	0.386

3 M, 3-month follow-up; 6 M, 6-month follow-up; 12 M, 12-month follow-up; NA, not applicable.

## Data Availability

Data is contained within the article.
